# Serum Oxidative Stress-related Biomarkers in Ocular Hypertension and Glaucoma

**DOI:** 10.18502/jovr.v19i4.15011

**Published:** 2024-12-31

**Authors:** Mine Esen Baris, Onur Furundaoturan, Meltem Kocamanoğlu, Seray Şahin, Yasemin Akçay, Suzan Güven Yılmaz

**Affiliations:** ^1^Ege University Hospital, Department of Ophthalmology, Izmir, Turkey; ^2^Ege University Hospital, Department of Medical Biochemistry, Izmir, Turkey; ^4^Mine Esen Baris: https://orcid.org/0000-0003-1341-6737

**Keywords:** Hypertension, Ocular, Open-angle Glaucoma, Oxidative, Stress

## Abstract

**Purpose:**

To evaluate the serum levels of oxidative stress-related molecules in patients with ocular hypertension (OHT) and primary open-angle glaucoma (POAG) compared with healthy controls.

**Methods:**

Treatment-naive patients with no known systemic diseases and with OHTand POAG diagnosis were recruited for the study. Also, age- and gender-matched healthy volunteers with no ocular and systemic diseases were included as controls. None of the participants were under any topical or systemic treatment or vitamin/antioxidant supplements. Smokers were excluded from the study. Serum levels of total antioxidant capacity (TAC), ascorbic acid, protein carbonyls (PCs), advanced glycation end products (AGEs), neuronal pentraxin 2 (NPTX2), and β -amyloid precursor protein(A β ) were analyzed.

**Results:**

A total of 90 patients (30 in each group) were included in the study. There was no statistically significant difference between the study groups in terms of age and gender distribution. Serum levels of TAC (1.47 ± 0.11 mmole/L) were significantly higher in patients with OHT compared to controls (1.40 ± 0.11 mmole/L) and patients with POAG (1.30 ± 0.08 mmole/L) (*P* < 0.05). However, there was no significant difference between the study groups in terms of serum levels of ascorbic acid, PCs, AGEs, NPTX2, and A β .

**Conclusion:**

Serum levels of TAC were significantly higher in patients with OHT. This elevated level might contribute to the protection of the optic nerve even in the presence of increased intraocular pressure.

##  INTRODUCTION

Glaucoma is the leading cause of irreversible blindness worldwide and is characterized by the neurodegeneration of the optic nerve, preceded by progressive retinal ganglion cell (RGC) death. Although increased intraocular pressure (IOP) is the only modifiable risk factor and, therefore, the only treatment target, many patients with progressive glaucoma have normal IOP values at diagnosis (normal-tension glaucoma) and many people with increased IOP show no signs of optic nerve damage (ocular hypertension [OHT]). Research reveals that, in addition to barotrauma, pathophysiological process of glaucoma also includes various factors such as autoimmunity, neuroinflammation, oxidative stress, and anatomical factors related to optic nerve head and lamina cribrosa. ${\;}^{\left[1--4\right]}$ The presence of multiple pathophysiological mechanisms could imply that there might be treatment targets other than increased IOP. The interactions between these mechanisms also complicates the identification of specific biomarkers for early detection of glaucoma.

OHT, on the other hand, is the condition in which IOP is high but optic nerve and visual field tests are normal. There is evidence supporting that some of these eyes develop glaucoma over time, but the majority do not show any damage in optic nerve. It is important to recognize which patients to treat versus those to monitor, but there is no specific parameter to use as a biomarker for this decision.

With both experimental and clinical studies, oxidative stress is shown to play a role in both initiating and accelerating glaucomatous RGC loss and optic neurodegeneration.^[5]^ The accumulation of oxidative stress-related end products in the trabecular meshwork (TM) seems to result from the combination of TM tissue malfunction in the conventional outflow pathway and the neuroinflammation process in the optic nerve head and RGCs.^[3]^ Therefore, oxidative stress-related molecules might serve as potential biomarkers for glaucoma. Various molecules have been suspected to play a role in the pathophysiology of glaucoma. Some of these molecules such as antioxidants have been identified as protective factors, while others have acted in the opposite direction to directly harm or exacerbate certain tissues. Total antioxidant levels or ascorbic acid levels have been considered as preventive factors or markers, whereas protein carbonyls (PCs) or advanced glycation end products (AGEs) are commonly associated with oxidative damage in various diseases. ${\;}^{\left[3--5\right]}$

β -amyloid precursor protein (A β ) is a peptide accumulated in different tissues such as ganglion cell layer and optic nerve in patients with glaucoma or other diseases such as diastolic dysfunction, myositis, various cancers, and Alzheimer disease. By its accumulation, A β is believed to contribute to glaucomatous damage. Another molecule called neuronal pentraxin 2 (NPTX2) is also a novel biomarker for Alzheimer disease, but no study has yet investigated its serum or aqueous humor levels in patients with glaucoma.

This study aimed to analyze the levels of some of the serum oxidative stress-related biomarkers, in addition to A β and NPTX2, in patients with OHT and compare the results to those from patients with primary open-angle glaucoma (POAG) and healthy controls.

##  METHODS

This prospective study was conducted at the Ophthalmology Department of Ege University Medical Faculty Hospital in Izmir, Turkey. Participants included patients diagnosed with OHT or POAG who were under treatment and/or clinical follow-up for at least six months between March 2022 and August 2023. Also, patients who applied for cataract surgery without any other ophthalmological pathologies were included as healthy controls. The study was approved by the Ethics Committee of the aforementioned hospital (decision no:21-4T/49), and written informed consent was obtained from all participants.

### Patients

**Figure 1:**
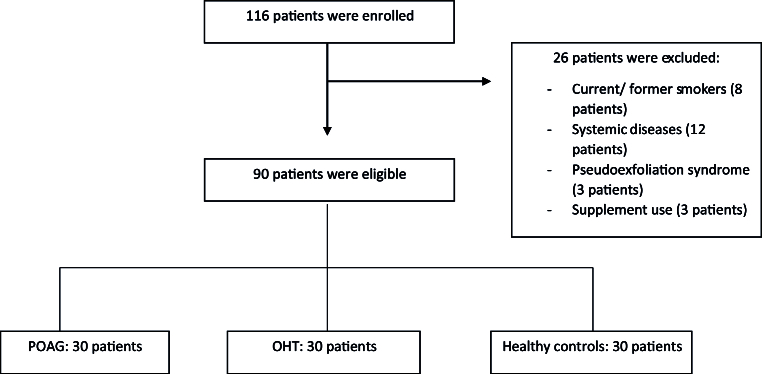
Flowchart of patient enrollment..

The recruited patients were aged between 45 and 70 years old, with no known systemic diseases (including neurodegenerative diseases, diabetes mellitus, arterial hypertension, and heart disease) and no history of any major surgeries (including cardiac and pulmonary procedures). Besides, they were not under any systemic treatment for any type of disorders and did not have any ocular diagnosis other than OHT and POAG. The control group consisted of participants with no ocular pathologies other than cataract. All participants were non-smokers. On the other hand, we excluded patients younger than 45 years old, those with < 6 months of follow-up, current or former smokers, and individuals with systemic diseases or undergoing systemic treatment (including antioxidant/vitamin supplements). Patients who had any other ocular pathologies (including pseudoexfoliation), those who started any systemic medications during follow-up, or those who had a visual acuity < 20/40 in either eye were also excluded [Figure 1].

All patients underwent complete ophthalmological examinations which included:

• best corrected visual acuity (BCVA),

• IOP measured with Goldmann applanation tonometer

• iridocorneal angle assessment using single mirror goniolens 902 (Haag-Streit, Koeniz, Switzerland),

• bio-microscopic anterior segment and fundus examination using 90 D lens

• visual field test based on the 24-2 SITA standard using Humphrey visual field analyzer (Carl Zeiss Meditec, Dublin, CA)

• peripapillary retinal nerve fiber layer thickness measurement using optical coherence tomography (Triton Swept-Source, Topcon, Tokyo, Japan).

It should be mentioned that eyes with image quality under 7 for optical coherence tomography (OCT) were excluded from the study.

OHT diagnosis was based on the following criteria:

• IOP measurements > 21 mmHg in at least two different examinations with a minimum interval of two weeks

• open iridocorneal angle on gonioscopy

• at least two normal and reliable visual field tests

• normal optic disc appearance on clinical examination

• normal peripapillary nerve fiber layer analysis, showing no progression for at least six months.

POAG was diagnosed based on glaucomatous visual field defects observed in at least two tests, characteristic glaucomatous appearance of optic disc with or without increased IOP, in addition to normal anterior chamber and gonioscopically open iridocorneal angle. All patients had moderate to advanced stage glaucoma with no history of glaucoma surgery or laser treatment. Patients with mild glaucoma were excluded to achieve a more homogenous study group.

Both eyes of each participant were required to meet the eye-specific criteria for inclusion in the study, and the eye with the better-quality imaging data was considered for statistical analysis.

### Blood Analyses

For blood sampling, the patients were asked to visit the clinic between 10:00 AM and 12.00 PM after abstaining from any physical exercise for at least 24 hours to minimize any potential interference with the results. All individuals were enrolled from same region of the study country in order to ensure a relatively homogeneous dietary intake consistent with Mediterranean dietary habits.

Blood serum samples collected from the patients were used for biochemical analysis. Disposable vacuum blood collection tubes with a gel separator were used for blood sample collection. Blood samples were collected from each patient, and the tubes were immediately centrifuged at 2000 × g for 10 minutes at +4ºC after blood clot formation. Also, 1 ml of serum sample was transferred from each sample to a microcentrifuge tube labeled with the patient's identifier and stored at –80ºC until the biochemical analyses were performed.

Total protein concentrations were measured with 25 µL of serum sample by the bicinchoninic acid assay kit (Bio Basic Inc., Markham, Canada) a colorimetric-based technique. The levels of A β PCs, NPTX2, and AGEs were measured by enzyme-linked immunosorbent assay (ELISA) kit (BT LAB, China) using 40 µL serum sample. Total antioxidant status was measured using 18 µL of serum sample by Trolox equivalent antioxidant capacity assay (TEAC) kit (Rel Assay Diagnostics, Gaziantep, Turkey), a colorimetric-based technique. All measurements were conducted according to the manufacturers' instructions.

### Statistical Analysis

Data were analyzed using the SPSS version 25.0 (IBM Corp., Armonk, New York). The clinical baseline data were analyzed using descriptive statistics, including mean, standard deviation, median, minimum, maximum, and percentage values. The Shapiro-Wilk test was used to check the normality assumptions of the quantitative data. Also, one way ANOVA with Bonferroni test for post hoc analysis was used for comparing the means between the three groups. Statistical significance was defined as *P* < 0.05.

The standardized effect size was calculated using the *t*-test table values, comparing the total antioxidant capacity (TAC) between the control group and the POAG group.^[6]^ Accordingly, considering 95% power, 1.03 effect size, and a type 1 error level of 0.05, it was determined that a minimum of 21 individuals per group would be required for this study. The sample size was calculated using G*Power 3.1.9 program.^[7]^

##  RESULTS

Totally, 90 (47 female, 43 male) patients were included in the study. The mean age of all patients was 64.2 ± 9.7 (range, 43–78) years. The study sample comprised 30 patients with OHT, 30 patients with POAG, and 30 healthy participants as controls. The mean follow-up period was 16.9 ± 10.1 (range, 6–48) months. All three groups were similar in terms of mean age and gender distribution. Demographic data and ophthalmic features of all studied eyes are displayed in Table 1.

TAC, ascorbic acid, PCs, AGEs, NPTX2, and A β levels of all groups are summarized in Table 2. The results showed that only TAC levels had a statistically significant difference among the three groups. Also, no significant relationship was found in terms of gender or glaucoma stage in subgroup analyses.

**Table 1:** Demographic data and ophthalmic characteristics of all study eyes.

**Feature**	**OHT ${\;}_{1}$ **	**POAG ${\;}_{2}$ **	**HCs ${\;}_{3}$ **	**P ${\;}_{1}{\;}_{-}{\;}_{3}$ **	**P ${\;}_{1}{\;}_{-}{\;}_{2}$ **	**P ${\;}_{2}{\;}_{-}{\;}_{3}$ **
Number of patients, *n*	30	30	30	–		–
Number of eyes, *n*	60	60	60	–	–	–
Age, yrs, mean ± SD	63.6 ± 8.7	63.9 ± 10.4	65.2 ± 10.1	0.8	1	0.8
Gender, *n *(%)						
- Female	16 (53.3)	16 (53.3)	15 (50)	0.9	1	0.9
- Male	14 (46.7)	14 (46.7)	15 (50)	0.9	1	0.9
Intraocular pressure, mmHg, mean ± SD	26.3 ± 5.3	26.6 ± 9.0	15.8 ± 4.1	0.7	0.02	0.02
Axl, mm, mean ± SD	23.4 ± 2.5	23.6 ± 2.1	23.3 ± 2.4	0.8	0.9	0.8
c/d ratio, mean ± SD	0.4 ± 0.1	0.8 ± 0.3	0.3 ± 0.5	0.6	0.01	0.01
RNFL thickness, µm, mean ± SD	103.8 ± 6.2	75.8 ± 18.3	105.6 ± 4.4	0.02	0.003	0.003
OHT, ocular hypertension; POAG, primary open angle glaucoma; HCs, Healthy controls; Axl, axial length; RNFL, retinal nerve fiber layer						

**Table 2:** Serum levels of oxidative stress-related molecules, neuronal pentraxin 2, and β -amyloid precursor protein in all study eyes.

**Molecule**	**OHT ${\;}_{1}$ **	**POAG ${\;}_{2}$ **	**HC ${\;}_{3}$ **	**P ${\;}_{1}{\;}_{-}{\;}_{2}$ **	**P ${\;}_{1}{\;}_{-}{\;}_{3}$ **	**P ${\;}_{2}{\;}_{-}{\;}_{3}$ **
Total anti-oxidant capacity (mmole/L)	1.47 ± 0.11	1.30 ± 0.08	1.40 ± 0.11	0.000	0.005	0.04
Protein carbonyls (ng/ml)	135.7 ± 93.3	169.4 ± 176.7	147.8 ± 94.2	0.06	0.1	0.08
Advanced glycation end products (ng/L)	265.7 ± 34.9	298.2 ± 120.7	273.2 ± 40.9	0.08	0.2	0.1
Ascorbic acid (mg/dL)	0.8 ± 0.2	0.7 ± 0.3	0.8 ± 0.1	0.9	0.8	0.9
Neuronal pentraxin 2 (nd/ml)	2.4 ± 3.2	2.6 ± 5.8	1.6 ± 4.5	0.9	0.6	0.4
Beta amyloid precursor protein (ng/ml)	9.7 ± 11.7	8.9 ± 13.7	9.5 ± 9.5	0.5	0.8	0.6
OHT, ocular hypertension; POAG, primary open angle glaucoma; HC, healthy controls All values were given as mean ± SD						

##  DISCUSSION

Despite their strong association, OHT and POAG are two different entities. Although IOP is elevated in patients with OHT, ocular examination and functional and structural tests do not reveal any signs of optic nerve damage. On the other hand, POAG is also frequently—but not always—associated with elevated IOP, and there are signs of optic nerve damage that lead to irreversible loss of visual field and decreased visual acuity. Although elevated IOP is the major and only modifiable risk factor in POAG, evidence shows that only 36% of eyes with thin/normal central corneal thickness and only 13% of eyes with thicker corneas (central corneal thickness between 550 and 588 μ m) and high IOP will develop glaucomatous neurodegeneration within five years.^[8]^ There are charts and risk calculators for clinicians to determine which patients require IOP-lowering medications, but there is still no definite, single biomarker in clinical practice to guide treatment decisions.

Oxidative stress has been shown to play a role in the process of progressive RGC loss, which is characteristic of glaucoma. Both animal and human studies have reported an imbalance between antioxidant defense mechanisms and oxidative stress in glaucoma.^[3,9,10]^

Experimental models have shown that antioxidant treatment can decrease neuroinflammation in glaucoma.^[11]^ Some studies have reported that dietary antioxidant molecules like omega 3 or omega 6 fatty acids, coenzyme Q10, melatonin, vitamin B3, vitamin C, vitamin E, and various other natural compounds like coffee, tea, ginkgo biloba, coleus, tropical fruits might help both regulate high IOP and enhance neuroprotection.^[12]^

In this study, the serum TAC was significantly higher in patients with OHT, compared to controls and patients with POAG. However, there was no statistically significant difference in serum ascorbic acid levels between these groups. The serum levels of PCs and AGEs were highest in patients with POAG, followed by the control group and patients with OHT, but the differences was not statistically significant. It is possible, however, that studies with larger group of patients might reveal a significant difference.

Hondur et al reported that serum levels of AGEs and PCs were significantly higher in patients with POAG compared to healthy controls, however, the differences were more prominent in aqueous humor samples.^[13]^ Erdurmus similarly reported that serum levels of TAC were higher in healthy controls compared to patients with POAG, whereas serum levels of PCs were higher in patients with POAG compared to the control group.^[14]^ Engin et al reported that TAC and AGEs were both lower in patients with glaucoma compared to healthy controls.^[15]^

Antioxidant defense mechanisms decline with age, resulting in increased susceptibility of tissues and cells to oxidative damage. This might explain the role of oxidative stress and its association with lower ocular blood flow, which is observed in normal-tension glaucoma.^[16]^ However, it seems that additional mechanisms are also involved in relation to POAG. The altered aqueous humor outflow in POAG increases IOP, resulting in the activation of oxidative stress pathway as a secondary factor in the pathogenesis of POAG. Also, increased reactive oxygen species (ROS) will induce damage in TM and cause a reduction in outflow, resulting in elevated IOP.^[17,18]^

Experimental studies have reported that the rise in ROS starts immediately after an increase in IOP, which in turn induces an endogenous antioxidant defense system via a nuclear factor erythroid 2-related factor 2 (NRF2) pathway.^[19]^ Meanwhile, if elevated IOP induces the oxidative pathways and causes ROS and other mediators to both initiate and sustain neuroinflammation in glaucoma, the question arises as to why most eyes with OHT remain damage-free or at least maintain a healthy optic disc for considerably longer periods. The reasons could lie in anatomical differences or variations in oxidative stress pathways.^[20]^. Our findings demonstrated lower serum levels of oxidative stress end products and higher levels of TAC in patients with OHT, which together might have a protective impact on the optic nerve against elevated IOP.

A β is a peptide with anti-microbial activity, and it can be secreted from various types of cells such as vascular endothelial cells, neurons, and platelets.^[21,22,23]^Many studies have reported that A β , similar to Alzheimer disease, is implicated in glaucoma pathogenesis.^[24,25]^ Experimental glaucoma models have shown the accumulation of A β in retina, especially in RGCs; furthermore, application of synthetic A β had led to induced RGC death, and anti-A β treatment has prevented RGC apoptosis in patients with glaucoma.^[26,27]^

Cappelli et al evaluated the aqueous humor levels of A β in glaucoma and reported no significant difference between glaucomatous and healthy eyes.^[28]^ They included eyes with POAG, secondary open-angle glaucoma, and primary angle closure glaucoma. The diverse etiologies of glaucoma might influence A β levels. Our current study also demonstrated no significant difference in serum levels of A β , despite the fact that we evaluated homogenous groups. The reason for this discrepancy might lie in differences in terms of the origin of A β : while serum A β could originate in platelets, the A β accumulated in RGCs is likely derived from other sources.

NPTX2 is a member of the protein family (neuronal pentraxins). It is secreted from pyramidal neurons and is considered a biomarker for AD.^[29]^ Its levels are shown to decrease in Alzheimer disease, and it is a strong predictor of progression from mild cognitive impairment to Alzheimer disease.^[30,31,32]^ In the present study, although serum levels of NPTX2 were lower in the POAG group compared to healthy controls and the OHT group, the difference was not significant. To the best of our knowledge, this is the first study to evaluate NPTX2 levels in POAG or OHT. Further studies with larger sample sizes might help better understand the role of NPTX2 in glaucoma, if any. Also, analyzing aqueous humor levels instead of serum levels could provide more relevant insights in revealing any possible association between NPTX2 and glaucoma.

To the best of our knowledge, this is the first study to compare the differences in serum oxidative stress-related molecules between patients with elevated IOP with and without glaucoma.

In this study, we did not consider systemic risk factors associated with oxidative stress such as diabetes, cardiac or pulmonary diseases, and smoking, and the impact of aging on oxidative stress was minimized. There are other limitations in this study as well. This was a cross-sectional study with no prospective follow-up. Since a number of patients in the OHT group would eventually develop glaucoma, long-term data would provide more valuable insights. Another limitation of this study is the lack of specific data regarding the levels of individual markers in the aqueous humor. While the current study provides valuable insights into serum levels of TAC in glaucoma, the clinical application may be limited due to the lack of sufficient data on the concentrations of each marker present in the aqueous humor. Future studies incorporating quantitative analysis of these markers could further enhance the clinical relevance of our findings and provide more robust clinical implications.

Moreover, topical antiglaucoma drugs may induce systemic side effects even at relatively low dosages; however, significant systemic effects, particularly on the biomarkers we studied, are uncommon. To the best of our knowledge, literature pertaining to this subject is lacking and warrants further exploration in future studies.

In summary, the balance between oxidative stress and antioxidant defense mechanisms may contribute to protecting the optic nerve and RGCs from elevated IOP in patients with OHT.

##  Financial Support and Sponsorship

This study was supported by the Ege University Scientific Research Project Committee under Grant number: 23429.

##  Conflicts of Interest

None.
